# NH Compare’s Health Deficiency 5-Star Rating: Do We Learn Different Things From Survey and Complaint Deficiencies?

**DOI:** 10.1093/geroni/igaa057.129

**Published:** 2020-12-16

**Authors:** Lindsay Peterson, John Bowblis, Kathryn Hyer

**Affiliations:** 1 University of South Florida, Tampa, Florida, United States; 2 Miami University, Oxford, Ohio, United States

## Abstract

The Centers for Medicare and Medicaid Services (CMS) publishes a 5-star rating system for nursing homes (NH). Currently, the 5-star rating for health deficiencies weights deficiencies from annual recertification surveys and complaints equally. Complaint deficiencies may contain different information than survey deficiencies because complaint deficiencies originate with consumers and complaint inspections are less predictable than surveys. The objective of this study is to construct separate 5-star ratings for survey and complaint deficiencies, and to compare them to CMS’ 5-star rating for health deficiencies. Using CASPER and ASPEN Complaints/Incident Tracking System for all NHs in 2017 (N=15,373), we calculated the 5-star rating for health deficiencies as reported by CMS, and then decompose CMS’ rating into separate 5-star ratings for survey and complaint deficiencies. The overall distributions of the CMS’ deficiency rating and survey deficiency rating are similar. The distribution of the complaint deficiency rating is different from CMS’ deficiency rating. Using complaint deficiencies, more NHs have 5-stars (26.5% vs. 10.5%) and fewer facilities have 4-stars (11.2% vs. 23.3%). Comparing the ratings for each facility relative to CMS’ rating, 35.3% of NHs have a different survey deficiency rating while 54.4% have a different complaint deficiency rating. A 5-star rating based on survey and complaint deficiencies results in different ratings for NHs, indicating that complaint deficiency ratings contain different information from survey deficiency ratings. CMS should publish separate ratings based on survey and complaint deficiencies to provide different information.

